# Value of [^18^F]flurpiridaz PET/CT for preoperative localization of atypical parathyroid tumors in primary hyperparathyroidism

**DOI:** 10.1007/s00259-025-07487-6

**Published:** 2025-07-29

**Authors:** Thorsten Derlin, Frank M. Bengel, Dennis Kleine-Döpke, Moritz Schmelzle, Bastian P. Ringe

**Affiliations:** 1 Institute of Radiology, Nuclear Medicine, and Molecular Imaging, Heart and Diabetes Center North Rhine-Westphalia, Georgstr. 11, 32545 Bad Oeynhausen, Germany; 2 Department of Nuclear Medicine, Hannover Medical School, Hannover, Germany; 3 Department of General, Visceral and Transplant Surgery, Hannover Medical School, Hannover, Germany


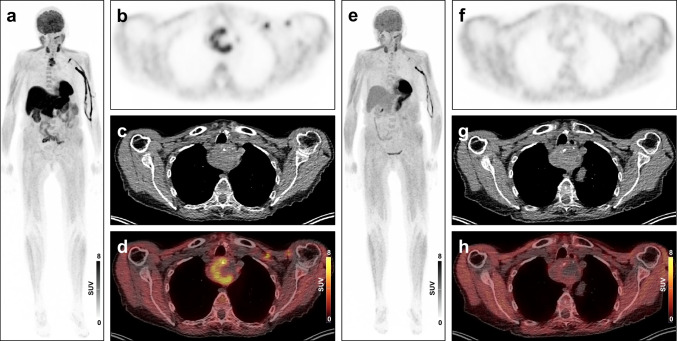


Primary hyperparathyroidism (pHPT) is a common endocrine disorder, most frequently caused by a solitary adenoma of the parathyroid glands, less frequently by multi-glandular parathyroid disease and rare types of parathyroid neoplasms [1, 2]. The feasibility of targeting mitochondrial complex I using [^18^F]flurpiridaz PET to localize mitochondria-rich parathyroid adenomas in pHPT has recently been demonstrated [3].

Here, we report the use of [^18^F]flurpiridaz PET/CT [4] for preoperative detection and localization of hyperfunctioning parathyroid tissue in a 78-year-old female patient after previous failure of surgical exploration. Intact parathyroid hormone was elevated (1096 ng/mL; normal values are 15 to 65 ng/mL) and the patient had hypercalcemia (3.39 mmol/L; normal values are 2.2 to 2.55 mmol/L), consistent with primary hyperparathyroidism. Preoperative ultrasound localization studies in another institution had suggested bilateral parathyroid adenoma at the lower poles, measuring up to 4 mm, but parathyroid adenoma was not confirmed during transcervical exploration. Subsequent dual-time-point [^18^F]flurpiridaz PET/CT (**a-d**) demonstrated intense uptake in a partially cystic space-occupying lesion in the upper mediastinum at 10 min p.i. (63 mm transversal diameter, SUVpeak 7.2), with uptake declining over time (SUVpeak 1.7 at 90 min p.i. (**e–h**)). At time of PET, intact parathyroid hormone remained elevated (609 ng/mL) and the patient had moderate hypercalcemia (3.07 mmol/L). Histopathological evaluation after surgical resection revealed an atypical parathyroid tumor with extension into the capsule. Following surgery, calcium levels declined to 2.09 mmol/L, and parathyroid hormone was nearly normalized upon discharge from the hospital (79 ng/mL).

Atypical parathyroid tumors are rare, accounting for < 2% of all parathyroid neoplasms [5], and represent a group of parathyroid neoplasms of uncertain malignant potential demonstrating atypical histological features. The observed value of [^18^F]flurpiridaz PET/CT for localization of hyperfunctioning parathyroid tissue beyond adenoma provides a rationale for further research exploring its usefulness in different types of parathyroid neoplasms.

## Data Availability

Data sharing not applicable to this article as no datasets were generated or analyzed during the current study.
